# Characterization of the Expression of Angiogenic Factors in Cutaneous Squamous Cell Carcinoma of Domestic Cats

**DOI:** 10.3390/vetsci9070375

**Published:** 2022-07-21

**Authors:** Erwin Kristobal Gudenschwager-Basso, Valentina Stevenson, Dan Phillip Sponenberg, Thomas E. Cecere, William R. Huckle

**Affiliations:** Department of Biomedical Sciences and Pathobiology, Virginia-Maryland College of Veterinary Medicine, Virginia Tech, Blacksburg, VA 24061, USA; kristoba@vt.edu (E.K.G.-B.); valents@vt.edu (V.S.); dpsponen@vt.edu (D.P.S.); tcecere@vt.edu (T.E.C.)

**Keywords:** VEGF-A, PLGF, VEGFR1, VEGFR2, KDR, Flt-1, angiogenesis, cutaneous squamous cell carcinomas, cat, feline

## Abstract

**Simple Summary:**

Squamous cell carcinoma (SCC) is a malignant skin cancer that affects domestic animal species and humans with similar characteristics. Our research seeks to understand the mechanisms by which SCC progression depends on the development of a new blood supply (angiogenesis) in the host. Here, we queried our archive of cat SCC tumor samples to measure expression of genes coding for angiogenic signaling proteins that can exist in closely related forms with distinct biological properties. We observed that, when compared to normal skin, SCC tissues contained a greater abundance of gene transcripts encoding a form of the growth factor PLGF, predicted to have an altered distribution in the body. Similarly, altered patterns of expression were observed for forms of the PLGF receptor Flt-1, which can modulate angiogenesis. Future studies will test the relationship between these gene expression changes and the severity of SCC in order to establish them as predictive biomarkers of SCC progression in individual patients.

**Abstract:**

Cutaneous squamous cell carcinoma (CSCC) is a common malignant skin cancer with a significant impact on health, and it is important to determine the degree of reliance of CSCC on angiogenesis for growth and metastasis. Major regulators of angiogenesis are the vascular endothelial growth factor (VEGF) family and their associated receptors. Alternative pre-mRNA splicing produces multiple isoforms of VEGF-A and PLGF with distinct biological properties. Several studies highlight the function of VEGF-A in CSCC, but there are no studies of the different isoforms of VEGF-A and PLGF for this neoplasm. We characterized the expression of three isoforms of VEGF-A, two isoforms of PLGF, and their receptors in cat CSCC biopsies compared to normal haired skin (NHS). Although our results revealed no significant changes in transcript levels of *panVEGF-A* or their isoforms, the mRNA levels of PLGF I and the receptors Flt-1 and KDR were downregulated in CSCC compared to NHS. Differences were observed in ligand:receptor mRNA expression ratio, with the expression of *VEGF-A* relative to its receptor *KDR* higher in CSCC, which is consistent with our hypothesis and prior human SCC studies. Immunolocalization in tissue showed increased expression of all measured factors and receptors in tumor cells compared to NHS and surrounding vasculature. We conclude that the factors measured may play a pivotal role in CSCC growth, although further studies are needed to clarify the role of angiogenic factors in feline CSCC.

## 1. Introduction

Squamous cell carcinoma (SCC) is a highly malignant neoplasm that arises from epidermal cells, inducing differentiation into keratinocytes usually producing keratin [1,2]. SCC can be classified based on the location of its origin as oral SCC, ocular SCC, or cutaneous SCC (CSCC). The last is among the most common cancers in domestic animals and the second most common cancer in white humans [3], with estimated human incidence having increased 50–200% in the United States and Canada over the last two decades [4,5,6,7]. This neoplasm produces a large medical and economic impact due to its local invasiveness, limited treatment approaches, and tendency to recur [7,8,9].

Prolonged solar radiation exposure (producing actinic keratosis), lack of skin pigmentation, sparse hair cover, and papillomavirus infection are the main factors associated with SCC tumor induction. Similarly to other cancers, the progression of actinic keratosis into SCC has been related to mutations in key genes involved in cellular pathways that control DNA repair, cell growth, survival, and motility [10,11], facilitating tumor growth, invasion, and metastasis [12,13,14]. A mutation in the p53 gene due to ultraviolet radiation exposure has been correlated with the upregulation of the angiogenic factor VEGF-A in SCC [15]. This gene is present in more than 90% of CSCC in humans as well as bovine, canine, feline, and equine SCC [16,17,18]. Reflecting on the similar molecular tumor progression mechanisms between humans and animal species, it is vital to understand the basic cell biology of this neoplasm, including its degree of reliance on angiogenesis for growth and metastasis. In this regard, naturally occurring CSCC in cats is an attractive model to study CSCC, including the more aggressive types such as head and neck carcinoma in humans [19].

**Figure 1:**
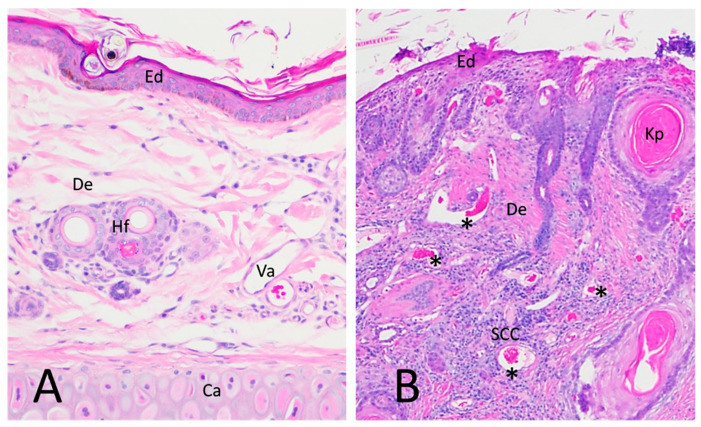
H & E microphotograph of normal haired skin (NHS) of the feline pinna and CSCC in a cat. (**A**) A surficial layer of flat keratin and multiple rows of intense basophilic keratinocytes form the epidermis (Ed). Deeper in the section is the dermis (De), with multiple hair follicles (Hf), small vasculature (Va), and adnexal glands, all distributed in a matrix of loose connective tissue. The lower part of the image has ear cartilage (Ca). (**B**) CSCC displaying invasive intradermal rafts of basophilic epithelial cells undergoing dysplastic and neoplastic keratinization (SCC), forming characteristic keratin pearls with a swirling glossy pink appearance (Kp). Intense proliferation of collagen fibers and blood vessels is present (*).

Histologically, CSCC in humans and cats is characterized by islands, cords, and trabeculae of unorganized epidermal keratinocytes, invading and trespassing across the basal layer of the epidermis into the dermis. Oftentimes, neoplastic keratinocytes form islands that secrete concentric eosinophilic keratin fibers in the center, forming keratin pearls that are useful for diagnosing SCC [20,21,22,23]. The tumor cells are normally large with an oval shape and have prominent hyperchromatic nuclei, although large degrees of anisocytosis and nuclear pleomorphism are described for less differentiated high-grade SCC. Tumor cells can have multiple desmosomes in the membrane, which are seen as bridges between cells [2]. CSCC locally invades the dermis and can reach bone and cartilage of affected areas with serious consequences for the patient. SCC induces an intense fibrous and inflammatory response from the surrounding tissue with abundant vascularization [24] (Figure 1). Naturally occurring SCC in cats shares multiple features with human cutaneous and head and neck squamous cell carcinoma; both are locally invasive, develop metastasis in advanced stages, often show local recurrence, and have similar tumor progression [19,25], making feline SCC a desirable animal model of Human SCC [19,24,25,26].

Angiogenesis is a major contributor to cancerous tumor growth, metastasis, and survival [27]. Solid tumors rely on vascular perfusion to grow and migrate; otherwise, they are limited to 1 to 2 mm before hypoxia in the center of the tumor induces necrosis [28]. Furthermore, measurement of microvascular density in tumors can be regarded as a reflection of angiogenic activity and used as a prognostic indicator for overall survival for lung and breast cancer, hepatic and gastric carcinoma, skin melanoma, and glioblastomas in humans [29,30,31,32,33,34,35]. Angiogenesis in normal and tumor tissue is stimulated by secreted peptides from tumor or adjacent stromal cells [36]. The vascular endothelial growth factor (VEGF) family, including VEGF; placental growth factor (PLGF); and their receptors Flt-1, sFlt-1, and KDR (also known as VEGFR-1, sVEGFR-1, and VEGFR-2, respectively), are the most important promoters of physiological and tumor angiogenesis [37,38,39,40,41,42,43,44,45,46].

VEGF-A interacts with higher infinity with Flt-1, although KDR induced stronger phosphorylation signaling cascade to induce proliferation, survival, and increase permeability in endothelial or tumor cells [47]. In contrast, PLGF interacts preferentially with Flt-1 and sFlt-1, indirectly controlling VEGF-A availability to interact with KDR with synergic effects [48,49,50]. Soluble Flt-1 lacks transmembrane and intracellular domains; thus, it is secreted to the extracellular space where it can act as a decoy receptor retained on the extracellular matrix, inducing an antiangiogenic effect [50,51]. Elevated expression of VEGF-A and its receptors has been linked with histopathology grading, tumor progression, and prognosis of multiple human tumors [52,53]. Similarly, the relative abundance of PLGF has been related to tumor vascularization and progression. Higher PLGF mRNA or protein levels correlate with pathological angiogenesis [46], tumor size, metastasis, advanced clinical stage, rate of recurrence, and poor prognosis of multiple types of cancers [54,55,56,57,58,59].

In cats, alternative splicing of pre-mRNA transcripts produces at least four isoforms of *VEGF-A* and two isoforms of *PLGF* (Gudenschwager et al., manuscript in preparation). These isoforms correspond to the well-characterized splice variants described in humans, mice, and other species [60,61]. The protein variants, which differ in their inclusion of key polypeptide domains, exhibit distinct biological behaviors associated with their differential sites and timing of expression, affinity for extracellular matrix, and liberation by proteolysis [62,63,64,65]. Larger VEGF and PLGF isoforms interact with the extracellular space due to the presence of heparin/heparan sulfate binding domains; they accumulate in the extracellular matrix forming a reservoir of growth factor that can be mobilized via simple dissociation or proteolysis [66]. In contrast, smaller isoforms have potential angiogenic actions on distant endothelial cells due to limited extracellular matrix interaction and thus increased mobility [47,48,60,62,63,66,67,68,69]. Selective expression of VEGF-A variants has shown that smaller, diffusible isoforms are linked to increased vascular perfusion, producing long, tortuous vessels with a larger caliber and less branching. In contrast, longer heparin-binding isoforms produce smaller, relatively dense capillary networks with increased branching [70,71,72,73,74,75,76,77]. VEGF-A isoforms also influence blood vessel fate as venules or arterioles. In conditional deletion studies, mice expressing only *VEGF164/164* were healthy and had normal retinal vasculature, whereas *VEGF120/120* mice exhibited severe retinal vascular outgrowth and reduced arterial differentiation, and *VEGF188/188* mice displayed normal venular outgrowth but impaired arterial development [78]. Taken together, these findings suggest that regulation of differential isoform expression is critical for generating a microenvironmental profile of angiogenic factors that assures the differentiation of a functional vascular network.

While differential VEGF isoform expression has been studied in a variety of developmental contexts, much remains to be learned about its role in spontaneous diseases. Thus, it is of interest to investigate whether VEGF and PLGF expression in cancers such as CSCC favors one or several isoforms in a manner that can be related to disease progression. To our knowledge, this has not been reported previously in any feline neoplasia. This information could improve our understanding of tumoral angiogenesis and could help provide rationale for targeted therapies for SCC treatment.

The role of VEGF/PLGF family members as drivers of human SCC angiogenesis is unclear. In oral and eyelid SCC, intra-tumor microvascular density revealed higher microvascular density in SCC compared to normal oral mucosa or normal eyelid skin [79,80]. In contrast, other studies found no significant differences in VEGF-A expression in human oral SCC compared to epithelial dysplasia or normal gingiva [81,82]. Furthermore, treatment with the VEGF-A antagonist bevacizumab, alone or in combination with 5-fluorouracil chemotherapy, failed to inhibit human oral SCC cell proliferation in vitro [83]. With regard to PLGF, transcripts and protein levels were upregulated in human oral SCC compared to normal tissue. Moreover, PLGF serum levels measured by ELISA were significantly correlated with advanced progression and poorer prognosis of oral SCC [59,84,85]. These conflicting results highlight the need for further research to clarify the role of these angiogenic factors in spontaneously occurring SSC.

In dogs, VEGF-A was immunodetected in SCC tumor tissue; particularly elevated levels of VEGF-A were found in SCC of the toe, a location that is typically more malignant and metastatic. However, VEGF-A was not elevated in other non-malignant neoplasms of the skin [86]. Additionally, intratumor microvascular density in canine CSCC displays a significant increase compared to trichoepitheliomas, a benign skin neoplasm [87]. These results suggest that VEGF-A could be a useful biomarker for evaluating malignancy in skin tumors of dogs. In feline SCC, there is scarce information about VEGF family expression and how it may influence tumor progression and tumoral angiogenesis. One study found a higher microvascular density of SCC located in the tongue compared to the ones in the mandibula or maxilla of cats [88], potentially explaining the clinical differences of poor clinical outcome of oral SCC in this location. Furthermore, transcripts encoding the lymphangiogenic factor VEGF-C were reported to be upregulated in feline oral and cutaneous SCC compared to normal control tissue [89]. In another study, VEGF immunolocalized in cutaneous SCCs was higher than in non-cutaneous tumors, although no significant correlation was found between tumor grading and VEGF-A expression. Additionally, VEGF-A was not detected in normal skin keratinocytes [90].

In the current study, we aimed to characterize the expression of mRNAs encoding three isoforms of VEGF-A (181, 163, 119 amino acid forms); two isoforms of PLGF (150 and 129 amino acid forms); and receptors for these growth factors (Flt-1; sFlt-1; KDR) in cat CSCC biopsies. We predict that pro-angiogenic factors are present in elevated levels in CSCC compared to expression in normal haired skin (NHS) and therefore may play a role in promoting tumor angiogenesis and growth. Knowledge of the role of these agents in tumor progression will contribute to our understanding of neovascularization mechanisms in feline SCC and help assess the utility of the feline disease as a model to study SCC in humans.

## 2. Materials and Methods

### 2.1. Sample Collection Selection and Preparation

Samples of CSCC were selected from the tissue archives of the Virginia Tech Animal Laboratory Services (ViTALS; an accredited diagnostic facility at the Virginia Maryland College of Veterinary Medicine), stored as formalin-fixed, paraffin-embedded (FFPE) blocks. Criteria of selection included cat breed; sex; the location of the tumor; and quality of the samples in terms of the size of the tumor, the relative area of tumor compared to normal tissue, time of storage, and good conservation of tissue. All cases selected were domestic shorthair cats, spayed female or neutered males having CSCC located in the pinna; cases were not segregated by degrees of ultraviolet light exposure. Hematoxylin and eosin (H & E)-stained 5 um sections were evaluated by a board-certified veterinary anatomic pathologist to assess the quality of the samples, evaluate the characteristics of the tumor (Figure 1B), and rule out the presence of other lesions of the skin (such as excessive dermatitis or necrosis of the tissue or the presence of parasites or fungi, any of which could confound our study). After careful examination, 14 cases dating from 2014 to 2018 were selected. Ten samples from normal skin to be used as controls were obtained from a local veterinary spay–neuter clinic, in the form of freshly discarded tips of pinna routinely excised to mark feral cats that had undergone surgical sterilization. Control skin tissue destined for histology was immediately placed in 10% formalin for 48 hrs and embedded in paraffin using ViTALS standard histological protocols. H & E-stained 5 um sections of normal skin were evaluated by a pathologist to reconfirm the absence of dermal lesions (Figure 1A).

### 2.2. RNA Purification and cDNA Synthesis

Total RNA was extracted from selected CSCC and normal skin FFPE blocks using the Quick-RNA FFPE Kit (Zymo Research, Irvine, CA, USA) following the manufacturer’s instructions. Briefly, 12 paraffin scrolls with a thickness of 5 um each were cut from individual blocks and placed in 1.5 mL Eppendorf tubes until further processed. For positive controls and qRT-PCR protocol development, total RNA was extracted from feline placental tissue using Trizol reagents (Invitrogen, Carlsbad, CA, USA) according to the manufacturer’s instructions with an additional DNase digestion using the Quick RNA mini prep kit (Zymo Reseach, Irvine, CA, USA) (Gudenschwager et al., manuscript in preparation). RNA concentrations were determined with a NanoDrop ND-100 Spectrophotometer (NanoDrop Technologies, Wilmington, DE, USA). Additionally, RNA quality was assessed in a representative group of samples using Agilent 2100 Bioanalyzer (Agilent Technologies, Palo Alto, CA, USA). Random-primed cDNA was produced from 1 µg of total RNA using High-Capacity cDNA Reverse Transcription Kit (Applied Biosystems, Foster City, CA, USA) in a 20 µL reaction according to the manufacturer’s instructions. cDNA was not diluted prior to use for PCR

### 2.3. Real-Time Quantitative PCR

**Figure 2:**
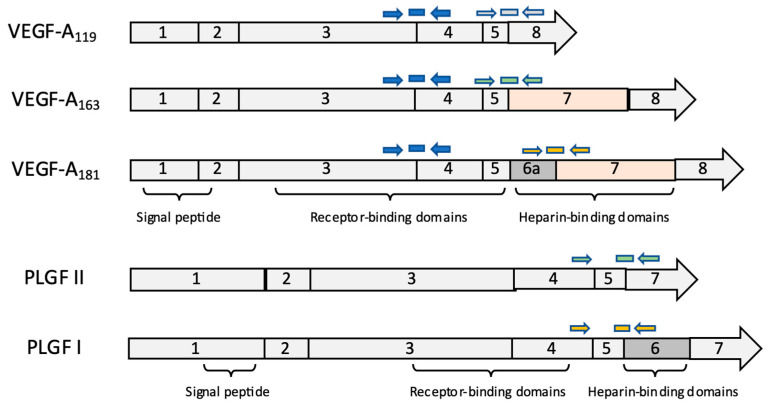
*VEGF-A* and *PLGF* isoforms. VEGF-A and PLGF occur in multiple protein isoforms, encoded by alternatively spliced pre-mRNAs that differ in their biochemical and biological characteristics. *VEGF-A* and *PLGF* exons 1–5 are present in all isoforms and code for the signal peptide and receptor-binding domains; splice variants exclude sequences encoding heparin-binding domains. The common and isoform-specific sites targeted by qRT-PCR are indicated by the arrows and bars, representing primers and fluorogenic probes, respectively.

DNA primers and minor groove binding (MGB) TaqMan DNA probes were designed using Primer Express 3.0.1 software (Applied Biosystems, Foster City, CA, USA) to target the exon–exon junctions unique to the respective *VEGF* and *PLGF* isoforms of interest (Table S1, Figure 2) as well as for full-length (signaling-competent) *VEGF* receptors *KDR* and *Flt-1* and the secreted decoy receptor *sFlt-1* [91]. Custom probes (Applied Biosystems) and primers (Operon, Huntsville, AL, USA) were used together with TaqMan Gene Expression Master Mix (Applied Biosystems, Foster City, CA, USA). All real-time PCR reactions were run on a One-Step System (Applied Biosystems) in 10 µL triplicate reactions for each sample in 96 well plates. *18S rRNA* was used as a normalizer gene with TaqMan VIC Ribosomal RNA control (Applied Biosystems, Foster City, CA, USA). Triplicate Ct values were averaged and used to determine the relative gene expression of genes of interest by the comparative Ct method [92]: for each sample, mean Ct values for each target were first normalized internally to 18S rRNA expression (yielding ∆Ct), then compared to the same target’s mean normalized expression in NHS controls (yielding ∆∆Ct). In some cases, the initial internal normalization was conducted using one of the genes of interest to examine the relationship between expression of functionally related mRNA species (e.g., PLGF II:PLGF I). For display, results were calculated as a fold change relative to mean expression in NHS (2^−^^∆∆Ct^). Two cDNA positive controls from feline placental tissue were included on each plate. Two negative control samples were used with ultrapure water to test for contamination, and two genomic (non-reverse transcribed) DNA controls were used in each qPCR run to confirm the cDNA dependence of the signal.

To verify the selectivity of isoform-directed qPCR assays, each isoform of feline PLGF and VEGF-A was cloned using sequence-specific primers spanning the entire coding regions and the pCR™2.1-TOPO System (Thermofisher Scientific, Waltham, MA, USA) (Gudenschwager et al., manuscript in preparation). cDNA dilution curves were generated to assess efficiencies of amplification, and probe and primer concentrations were adjusted to achieve >90% efficiency for each reagent set. The specificity of the isoform-directed reagents was confirmed by cross-reaction standard curves. For example, primers and probes developed for feline *VEGF-A_119_* were tested for their ability to detect known standard *VEGF-A_163_* and *VEGF-A_181_* cDNAs.

### 2.4. Immunohistochemistry (IHC)

Tissue sections from CSCC and NHS were cut at 5 μm, mounted, dried at 42 °C overnight, and stored at room temperature for less than 3 days before staining. All steps were performed at room temperature unless otherwise specified, using the ultraView Universal Alkaline Phosphatase Red Detection Kit from Ventana (cat. no. 760-501) and manual conventional histology protocols. Briefly, sections were deparaffinized in xylene, rehydrated, and washed with 1X reaction buffer (1X RB; Ventana cat. no. 950-300). Unmasking of antigens was performed in a polyethylene staining jar with Ventana cell conditioner 1 (950-124) for 60 min at 95 °C. Sections were then rinsed in 1X RB and blocked with 125 µL of Ventana 760-050 for 8 min. Primary antibody was diluted in Ventana incubation diluent (251-018); 125 µL of this dilution was used per slide (Table 1). Slides were incubated for 1 h and then washed with 1X RB. Negative control slides were incubated without primary antibody and only using incubation diluent. For secondary incubation, we used 125 µL of ultraView Universal Alkaline Phosphatase Red Detection Kit (Ventana 760-501), followed by 125 µL of UV red enhancer, incubated for 4 min. Then, 100 µL of UV Fast Red A and 100 µL of UV red Naphthol were applied and incubated together for 8 min. Finally, 125 µL of UV Fast Red B was incubated for 8 min in rinsed with 1X RB. Slides were counterstained with hematoxylin for 45 s, rinsed, and air-dried for at least 30 min before the coverslip was applied. Samples were observed under a Nikon Eclipse Ci-S microscope; images were captured on a Nikon camera and analyzed using NIS-Elements Analysis D 5.01 image software, Nikon Instruments Inc. (Melville, New York, NY, USA).

Feline cross-reactivity of the anti-human sequence antibodies used to detect VEGF-A and Flt-1 was confirmed by western immunoblotting of extracts from HEK293 cells overexpressing the feline proteins (Gudenschwager et al., manuscript in preparation). This finding was anticipated based on the high degree of sequence identity between human and feline VEGF-A and Flt-1 (92% and 86%, respectively) within the relevant peptide immunogens for these antibodies. The anti-PLGF antibody used was prepared against a human-sequence N-terminal peptide (found in all PLGF splice variants) with which the feline sequence bears 94% amino acid identity (46/49 residues) and 100% conservation. Similarly, human and feline KDR sequences share 93% identity (28/30 residues) in the peptide immunogen used by the vendor.

### 2.5. Quantification of IHC Staining

Photomicrographs of 100× magnification images of normal skin pinna from two cats and CSCC from the pinna of three cats were analyzed using the NIH software ImageJ 1.5j8 to determine the surface area of positive immunoreactivity on each image, allowing comparison of normal skin to CSCC. Specifically, a selection of red chromogen staining pixels, indicative of positive immunostaining, were located and selected using the color threshold tool. Specific ranges of hue 210–255, saturation 95–230, and brightness 190–255 were used for all images. The selected pixels were compared to the total number of pixels in the image to obtain the percentage of selected pixels as described previously by Jensen [93] and commonly found in the field [94,95,96]. Each sample was evaluated in three different randomly selected fields within the same tumor slide.

### 2.6. Statistical Analysis

Unpaired two-sided Student’s *t*-tests were used to evaluate differences in gene expression and IHC parameters between CSSC and normal skin using Graph pad Prism 9.1.0. Alpha = 0.05.

## 3. Results

For this study, 24 formalin-fixed paraffin-embedded (FFPE) samples were selected: 14 samples of CSCC biopsies from the ViTALS archive and 10 samples of NHS. cDNAs derived from total RNAs extracted from paraffin blocks were subjected to qPCR using cat-directed primers and probes. Protein localization in tissue was performed by IHC analysis using antibodies directed against VEGF-A, PLGF, Flt-1, and KDR in three FFPE tissue sections of CSCC and compared against normal skin.

### 3.1. Gene Expression Analysis

**Figure 3:**
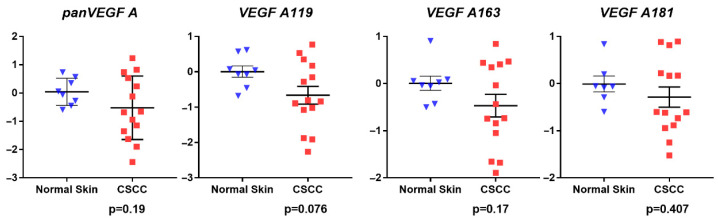
*VEGF-A* relative gene expression. mRNA expression of *panVEGF-A* and the indicated splice variants was estimated by qRT-PCR in cDNAs derived from FFPE sections of NHS and CSSC tissue. Results are shown as relative to mean 18S rRNA-normalized expression in NHS controls, calculated using the ΔΔCt method. Vertical axes represent expression in CSSC relative to NHS on a log_10_ scale. Blue triangles represent normal skin samples; red squares represent CSCC samples. *p* values are from two-sided unpaired Student’s *t*-tests.

**Figure 4:**
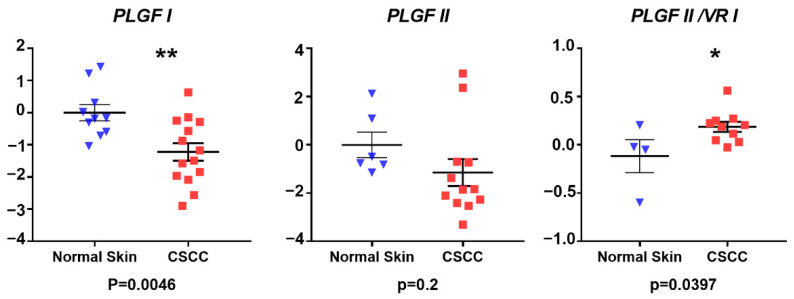
PLGF relative gene expression. mRNA expression of PLGF I and PLGF II splice variants was estimated by qRT-PCR in cDNAs derived from FFPE sections of NHS and CSSC tissue. Results are shown for the individual growth factors as relative to mean 18S rRNA-normalized expression in NHS controls, calculated using ΔΔCt method; similarly, PLGF variant ratios were calculated by internal normalization to PLGF I. Vertical axes represent expression in CSSC relative to NHS on a log_10_ scale. Blue triangles represent normal skin samples; red squares represent CSCC samples *p* values are from two-sided unpaired Student’s *t*-tests.

qRT-PCR analysis revealed similar mRNA expression levels of all *VEGF-A*-encoding species measured, including *panVEGF-A* (*p* = 0.19), *VEGF-A_119_* (p0.076), *VEGF-A_163_* (*p* = 0.17), and *VEGF-A_181_* (*p* = 0.407) in CSCC compared to NHS, although for each VEGF target, expression trended downward in CSCC (Figure 3). Comparison of PLGF isoform mRNAs in CSCC versus NHS revealed lower expression in CSCC that reached statistical significance for PLGF I (*p* = 0.0046) but not PLGF II (*p* = 0.2). Thus, the anticipated increase in angiogenic factor mRNA expression was not observed. Interestingly, however, when expression of PLGF II was normalized internally to PLGF I, a statistically significant increase in PLGF II:PLGF I mRNA expression ratio was noted in CSCC (*p* = 0.039) (Figure 4).

**Figure 5:**
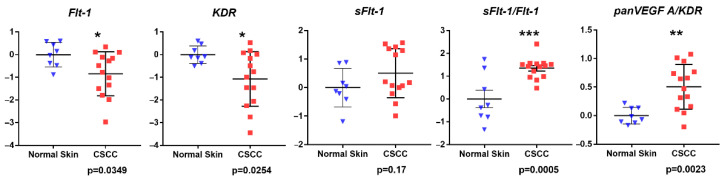
Relative expression of *VEGF/PLGF* receptor mRNAs. Relative expression of *Flt-1*, *sFlt-1*, and *KDR* mRNs was estimated by qRT-PCR in cDNAs derived from FFPE sections of NHS and CSSC tissue. Results are shown as relative to mean 18S rRNA-normalized expression in NHS controls, calculated using ΔΔCt method; similarly, *Flt-1* variant or *panVEGF-A*:*KDR* ratios were calculated by internal normalization to expression of full-length *Flt-1* or *KDR*, respectively. Vertical axes represent expression in CSSC relative to NHS on a log_10_ scale. Blue triangles represent normal skin samples; red squares represent CSCC samples. *p* values are from two-sided unpaired Student’s *t*-tests.

Expression of full-length forms of the *VEGF/PLGF* receptors KDR and Flt-1 were reduced (*p* = 0.0254 and *p* = 0.0348, respectively) in CSCC relative to NHS, whereas expression of the alternatively spliced decoy receptor *sFlt-1* was indistinguishable in tumor and control samples (*p* = 0.17) (Figure 5). However, estimating *sFlt-1* expression relative to its full-length counterpart *Flt-1* revealed a higher *sFlt-1:Flt-1* ratio in CSCC (*p* = 0.0005). Moreover, we detected differences in ligand/receptor expression ratios, as the expression of *panVEGF-A* relative to its principal angiogenic receptor *KDR* was likewise higher in CSCC (*p* = 0.0023).

### 3.2. Immunohistochemistry Results

**Figure 6:**
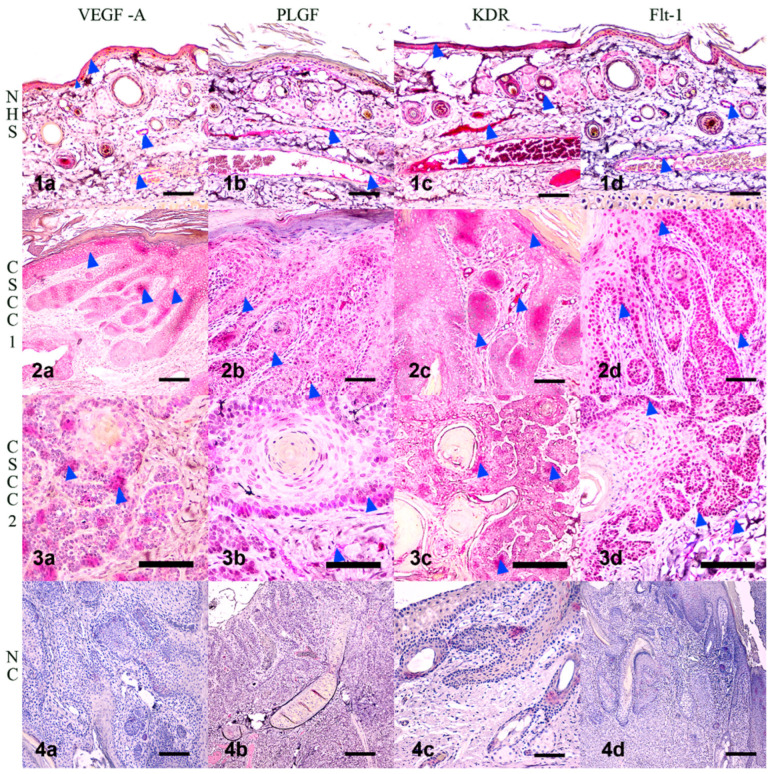
Immunolocalization of angiogenic biomarkers in NHS and CSCC. (**1a**) VEGF-A immunoreactivity in normal pinna in blood vessels, apocrine glands, and keratinocytes. (**2a**,**3a**) VEGF-A immunoreactivity in SCC, associated with neoplastic cells under hyperplastic and dysplastic keratinization. (**4a**) Negative control for VEGF-A (**1b**) PLGF immunoreactivity in the endothelium of blood vessels in the normal pinna. (**2b**,**3b**) Prominent immunoreactivity of PLGF in SCC keratinocytes of the stratum basale and accompanying the increased vascularization in the periphery of the tumor. (**4b**) Negative control for PLGF (**2c**,**3c**) KDR and Flt-1 immunolocalized in blood vessel endothelium and in SCC tumoral cells. (**4c**) Negative control for KDR (**1c**) Marked increase in immunoreactive KDR in CSCC compared to pinna NHS. (**1d**) Flt 1 immunoreactivity in apocrine glands and epithelium of blood vessels of NHS. (**2d**,**3d**) Flt 1 immunoreactivity localized to tumoral cells, especially in the stratum basale of the dysplastic epidermis in the periphery of dermal invaginations of tumoral keratinocytes (**4d**) Negative control for Flt-1. Blue arrowheads point to positive immunostaining. NHS—normal haired skin from pinna; CSCC—cutaneous squamous cell carcinoma; NC—Negative control omitting primary antibody. Scale bar represents 200 um.

IHC staining revealed that VEGF immunoreactivity in normal pinna was localized to blood vessels, apocrine glands, and keratinocytes. In CSCC samples, VEGF was found in neoplastic cells undergoing hyperplastic and dysplastic keratinization. VEGF immunoreactivity was also abundant in the vasculature around and inside the tumoral cells (Figure 6(1a,2a,3a,4a)). It is important to note that the localization of VEGF expression appeared to change between samples and even within the same section, in concordance with reports from other authors [97,98]. PLGF immunoreactivity was detected in the endothelium of blood vessels in the normal pinna. In CSCC samples, there was an apparent increase in PLGF immunoreactivity in neoplastic keratinocytes of the stratum basale and in the abundant vasculature in the periphery of the tumor (Figure 6(1b,2b,3b,4b)). KDR immunoreactivity was detected in blood vessel endothelium and in SCC tumor cells. There was a marked increase in KDR immunoreactivity in CSCC compared to the normal skin of the ear (Figure 6(1c,2c,3c,4c)).

**Figure 7:**
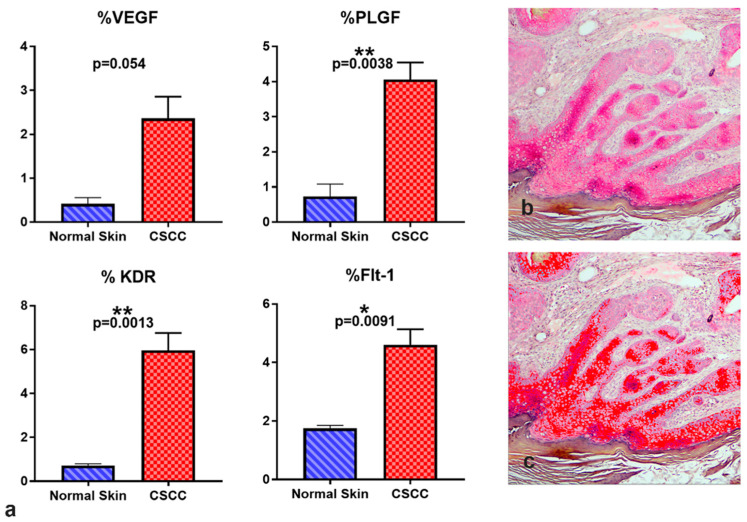
Quantification of angiogenic marker immunoreactivity in NHS and CSCC. (**a**) Using Image J, positive immunoreactive area was estimated as a percentage of total frame area for the indicated angiogenic biomarkers. (**b**) Representative image of pink Flt-1 immunoreactivity in CSCC. (**c**) Pixels flagged in red defined as Flt-1-positive by the image analysis of the same section.

Flt-1 immunoreactivity was localized in tumoral cells, especially in the stratum basale of the dysplastic epidermis and in the periphery of the invaginations of tumoral keratinocytes, showing a distribution similar to PLGF. Flt-1 immunoreactivity was also found in apocrine glands and epithelium of blood vessels (Figure 6(1d,2d,3d,4d)). To quantify the IHC signals in immunoreactive sites, Image J software was used to estimate the relative abundance of positive areas in histological images. Positive area per image is expressed relative to the total area of the frame, or percent of positive area. All proteins measured showed an increase in percent positive area in CSCC compared with normal skin of the pinna (Figure 7), although the highly variable VEGF-A immunoreactivity changes did not reach statistical significance (*p* = 0.054). These IHC results are consistent with a predicted increase in angiogenic protein presence in CSCC relative to control skin.

## 4. Discussion

The present study examined mRNA and protein expression of VEGF-A, PLGF, and their receptors KDR and Flt-1 in feline CSCC and NHS controls, testing the prediction that these mediators of tumor angiogenesis would be elevated in cutaneous carcinoma relative to normal skin. In addition, we measured expression of pre-mRNA splice variants of VEGF-A and PLGF, seeking evidence for altered profiles of growth factor isoform expression that may be associated with aberrant tumor neovascularization. Our results from protein immunolocalization experiments revealed that the abundance of immunoreactivity for ligands and receptors of the VEGF/PLGF family were increased in tissue sections of feline CSCC compared to NHS. These findings are consistent with increased PLGF reported in oral SCC [59,84] and increased VEGF-A and KDR in CSCC as described in dogs [86,87]. Overall, the IHC results are in accordance with the expectation that markers of angiogenesis are elevated in CSCC.

However, the increased protein immunoreactivity in CSCC contrasts with our gene expression results, which revealed that mRNA expression of VEGF and PLGF splice variants and their receptors was unchanged or moderately lower in CSCC relative to NHS. This discrepancy between observed expression patterns at the mRNA and protein levels may stem from a variety of factors. Possible mechanisms include increased mRNA translational efficiency or reduced rate of protein degradation in CSCC relative to NHS; either mechanism could contribute to elevation of observed protein abundance in the presence of relatively unchanged steady-state mRNA levels. Microenvironmental factors such as inflammatory cytokines, proteases, or nucleases could be in higher concentration in tumoral samples, thus predisposing these samples to RNA instability [99]. Tumor environment can affect transcriptional or translational regulation, with *cis*- and *trans*-acting mechanisms enhancing the synthesis of proteins from a relatively low abundant mRNA [100]. Other factors to consider include the increased endothelial Flt-1 and KDR protein presence that would naturally accompany increased vascular density in CSCC, and the fact that some of the angiogenic proteins of interest (VEGF-A, PLGF, and sFlt-1) may, as secreted and mobile elements, arrive in the field of IHC analysis after being expressed elsewhere [101]. In any event, the apparent disconnect between relative mRNA and protein expression suggests that measurement of mRNA levels alone may not be indicative of pro-angiogenic status in feline CSCC.

We detected a similar abundance of mRNAs encoding *PanVEGF-A* and its alternatively spliced isoforms in CSCC and NHS. *PLGF I* mRNA, as well as mRNAs encoding the VEGF family receptors *KDR* and *Flt-1*, were reduced in CSCC compared to normal skin, although *VEGF-A* relative to *KDR* was increased in CSCC. These results, although unforeseen, are in agreement with reports in humans where similar *VEGF-A* transcript levels were found in oral SCC compared to epithelial dysplasia or normal gingiva [81,82]. Furthermore, our results are in agreement with previous findings that protein immunoreaction against VEGF-A was reduced in head and neck SCC compared to precancerous lesions or to normal skin [102]. A reduction of VEGF-A immunostaining in cases of undifferentiated SCC compared to low-grade differentiated oral SCC was reported [103]. Similarly, VEGF-C immunodetection was described as reduced in feline cutaneous SCC compared to normal skin control [89]. Previous authors hypothesized that VEGF-A could participate in physiological functions in non-neoplastic tissue that is interrupted during neoplastic progression [97]. In contrast, our gene expression results differ from studies showing *VEGF-A* mRNA and *PLGF* upregulation in human oral SCC [59,84,104,105,106] and canine CSCC [86,87], although we report higher ratios of *panVEGF-A* relative to KDR in CSCC samples, suggestive of an abundance of ligand to receptor, thus favoring KDR activation within the tumor. We note that the presence of normal tissue around the tumor may contribute mRNA to that from the tumor and may explain the increasing abundance of *sFlt-1* relative to *Flt-1* in SCC.

In normal skin samples, VEGF-A, Flt-1, and KDR immunoreactivity was observed in blood vessels, apocrine glands, and keratinocytes, especially in the stratum basale of the epidermis, in agreement with other studies in human normal skin [97,107,108]. In CSCC samples, we found an apparent co-localization of VEGF-A, KDR, and Flt-1 in neoplastic keratinocytes of CSCC, suggesting an autocrine positive regulation to promote tumor growth and invasion, as has been postulated before for oral SCC [97,103]. In addition, we believe that *VEGF-A* and *PLGF* expression from neoplastic keratinocytes could be targeting endothelial cells to promote angiogenesis in the tumor. Based on these results, the VEGF family could play a crucial role in the tumoral progression of CSCC. We did not find differences in the patterns of expression of the *VEGF-A* splice variants between CSCC and NHS, leading us to conclude that the multiple isoforms of *VEGF-A* are working in concert to vascularize the tumor in a manner similar to that in physiological angiogenesis. However, the increased ratio of PLGF II:PLGF I noted in CSCC (Figure 4) suggests that a greater proportion of PLGF in CSCC may exist as the less matrix-associated/more mobile form. Similarly, the higher ratio of sFlt-1:Flt-1 in CSCC compared to NHS (Figure 5) is suggestive of a greater fraction of this VEGF-A/PLGF binder being available to serve as a local growth factor buffer. Overall, we can conclude that the factors measured may play a role in CSCC and that their associated paracrine or autocrine signaling cascades may favor tumor progression by increasing vascularity.

**Figure 8:**
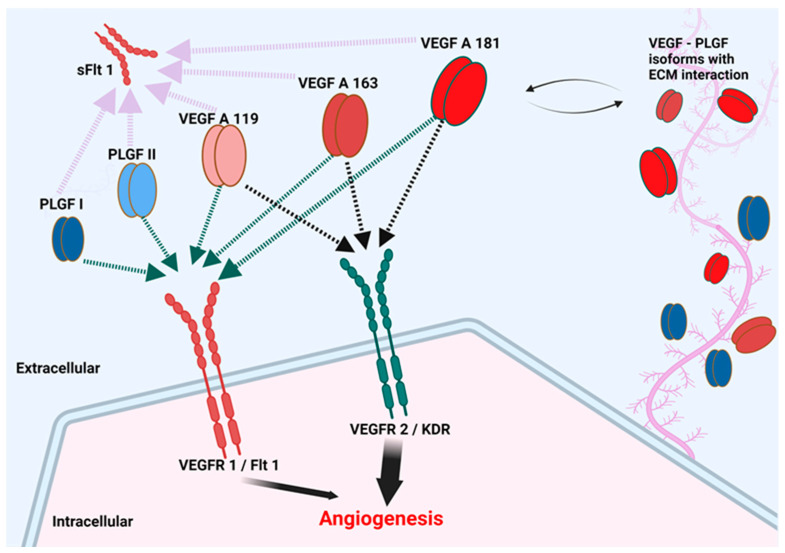
Isoforms of the VEGF-A family and their receptor interaction. Proangiogenic VEGF-A isoforms can bind both Flt-1/VEGFR1 and KDR/VEGFR2, but exert their principal angiogenic effects via KDR. In contrast, PLGF binds Flt-1 selectively and is able to compete with VEGF-A for Flt-1 binding, liberating VEGF to signal through KDR. PLGF II and VEGF-A_119_ lack extensive heparin-binding domains and therefore are relatively soluble in the extracellular space, while PLGF I, VEGF-A_163_ and VEGF-A_181_ interact with the extracellular matrix forming a reservoir in the extracellular matrix (ECM). sFlt-1, a truncated, secreted splice variant of Flt-1, binds ECM and thus can act paradoxically as a competitor for KDR signaling with antiangiogenic effects or as an accessible reservoir for VEGF-A. The ability of either PLGF or sFlt-1 to indirectly affect VEGF-A-stimulated angiogenesis by these mechanisms will depend on their distribution, which in turn are functions of their affinity for extracellular matrix as dictated by splice product dominance.

Limitations of this study include the possible presence of RNA contributed by marginal normal tissue in addition to neoplastic tissue in the same sample. A selection of the tumor from its nontumoral periphery in the FFPE slide would produce more tissue-selective results. In addition, we encountered high variability of measured transcript levels, especially in SCC samples, likely due in part to tumor heterogeneity associated with use of samples from outbred animal models in translational cancer research [109]. Nevertheless, our results encourage further studies into the possible role of VEGF, PLGF, and their receptors in mediating CSCC angiogenesis, progression, and metastasis, and accordingly, the utility of these agents as biomarkers for these critical events in disease. Of particular interest are the observed altered relationship between splice variants of Flt-1, the increased ratio of VEGF-A mRNA to that encoding its receptor KDR, and the apparent shift toward PLGF II over PLGF I in CSCC. The potential consequences of these changes in the tumor microenvironment include mobilization of the decoy receptor sFlt-1, greater saturation of the KDR receptor, and a relative increase in the less matrix-bound PLGF II isoform (Figure 8).

In the present work, we have explored CSCC in cats as a spontaneous disease setting in which to study angiogenic biomarker expression dynamics, with a focus on pre-mRNA splice variants. Although we did not observe the predicted changes in expression of mRNAs encoding individual heparin-binding or soluble VEGF-A variants in CSCC compared to normal skin, altered relationships among angiogenic growth factor mRNAs and those encoding their receptors emerged. These alterations (e.g., increases in PLGF II:PLGF I and sFlt-1:Flt-1 mRNA ratios) have in common a predicted consequence on the distribution of angiogenic mediators in the tumor microenvironment. Given the critical need for appropriate spatial and temporal deployment of angiogenic growth factors in normal vascular development [71,72,74,75,76,77], perturbation of the normal patterns may play a role in aberrant tumor vascularization. Future studies will test the relationship between these gene expression changes and the progression of SCC in order to more thoroughly assess their value as predictive biomarkers of SCC outcome in individual patients.

## Figures and Tables

**Table 1:** Antibodies used for IHC.

Protein Target	Antibody Name	Antibody Details	Dilution
panVEGF-A	C-1 (sc-7269) Santa Cruz	Mouse monoclonal IgG	1/100
PLGF	H-90 (sc-20714) Santa Cruz	Rabbit polyclonal IgG	1/100
KDR	F-10 (sc-393179) Santa Cruz	Mouse monoclonal	1/200
Flt-1	H-225 (sc-9029) Santa Cruz	Rabbit polyclonal IgG	1/200

## Data Availability

Data from gene expression analysis and quantification of IHC staining may be obtained from the corresponding author on request.

## Supplementary Materials

The following supporting information can be downloaded at https://www.mdpi.com/article/10.3390/vetsci9070375/s1. Table S1: List of primers and probes used for this study.
