# Supplementary material for: Characterization of the Expression of Angiogenic Factors in Cutaneous Squamous Cell Carcinoma of Domestic Cats
Source: Vet Sci. 2022 Jul 21;9(7):375. doi: 10.3390/vetsci9070375 (PMC9351683; doi:10.3390/vetsci9070375)
Supplement: Supplementary file 1 [file vetsci-09-00375-s001.zip › vetsci-1817279_Supplemental_Table_S1.pdf]

Supplemental Table S1: Primers and probes used for qRT-PCR

Target	Name	Strand	Sequence (5'-3')
PanVEGF A (exons 3/4)	BH-745	S	CCCACGGAGGAGTTCAACAT
	BH-746	AS	TGGCCTTGATGAGGTTTGATC
	BHTP-33	P	FAM-ACCATGCAGATTATGC-NFPMGB
VEGF-A$_{119}$ (exons 4/5/8)	BH-790	S	ATGTGAATGCAGACCAAAGAAAGA
	BH-791	AS	CACCGCCTGGGCTTGT
	BHTP-39	P	FAM-AGCGAAAGAAAAATGTGACAAG-NFPMGB
VEGF-A$_{163}$ (exons 4/5/7a/7b)	BH-711	S	AGCAAATGTGAATGCAGACCAA
	BH-712	AS	TGCAAGTACGTTCGTTTAACTCAAG
	BHTP-31	P	6FAM-AAAGAAAATCCCTGTGGGC-MGBNFQ
VEGF-A$_{181}$ (exons 6s/7)	BH-713	S	AATCAGTTCGAGGAAAGGGAAAG
	BH-714	AS	TGCGGATCTTGTACAAACAAATG
	BHTP-32	P	VIC-CAAGAAATCCCGTCCCTGT-MGBNFQ
PLGF I (exons 4/5/6)	BH-676	S	TCCTACGTGGAGCTGACATTCTC
	BH-674	AS	CCCTTGGGTCTCCTCCTTTC
	BHTP-27	P	FAM-CAGAAAGGAGGAGACCCA-MGB
PLGF II (exons 4/5/7)	BH-676	S	TCCTACGTGGAGCTGACATTCTC
	BH-679	AS	GTGGGGGTTGTCTCTCTTCTGA
	BHTP-28	P	VIC-AGAAAGGTGCGGCAAT-MGB
Flt-1 (exons 14/15)	BH-792	S	GGTTTAAAAACAACCACCAAATACAG
	BH-793	AS	TGCTGCTTCCCGGTCCTA
	BHTP-40	P	FAM-AACCCGGAATTATC-NFPMGB
sFlt-1 (exon 13/intron 13)	BH-794	S	CTGCAGAGCCAGGAACATATACA
	BH-795	AS	GATCCGAGAGAAAACAGCCTTTT
	BHTP-41	P	FAM-ACAATTAGAGGTGAGCACTG-NFPMGB
KDR (exons 3/4)	BH-751	S	CGTAGCCTCGGTCATTTATGTCT
	BH-752	AS	TGTTGGTCGCTAACAGAAGCA
	BHTP-36	P	FAM-TGTTCAAGATTACAGGTCTC-NFPMGB

S, Sense primer; AS, Anti-sense primer; P, Fluorogenic TaqMan probe
